# Concurrent Validity of Movement Screening Criteria Designed to Identify Injury Risk Factors in Adolescent Female Volleyball Players

**DOI:** 10.3389/fspor.2022.915230

**Published:** 2022-06-24

**Authors:** Sophia Ulman, Ashley Erdman, Alex Loewen, Michael Dressing, Charles Wyatt, Gretchen Oliver, Lauren Butler, Dai Sugimoto, Amanda M. Black, Joseph Janosky

**Affiliations:** ^1^Movement Science Lab, Division of Sports Medicine, Scottish Rite for Children, Frisco, TX, United States; ^2^Department of Orthopaedic Surgery, University of Texas Southwestern Medical Center, Dallas, TX, United States; ^3^Department of Orthopedics, Joe DiMaggio Children's Hospital, Hollywood, FL, United States; ^4^Sports Medicine and Movement Laboratory, School of Kinesiology, Auburn University, Auburn, AL, United States; ^5^Department of Rehabilitation, Nicklaus Children's Hospital, Miami, FL, United States; ^6^Faculty of Sport Sciences, Waseda University, Tokyo, Japan; ^7^The Micheli Center for Sports Injury Prevention, Waltham, MA, United States; ^8^Sport Injury Prevention Research Centre, Faculty of Kinesiology, University of Calgary, Calgary, AB, Canada; ^9^Sports Medicine Institute, Hospital for Special Surgery, New York, NY, United States

**Keywords:** injury prevention, risk reduction, anterior cruciate ligament injury, motion capture, agreement, youth sport, sports medicine

## Abstract

Anterior cruciate ligament (ACL) injuries in female adolescent athletes occur at disproportionately high levels compared to their male counterparts. However, limited prospective data exist on the validity of low-cost screening tools that can proactively identify ACL injury risk, specifically for female athletes. The purpose of this study was to assess the concurrent validity of a three-task injury risk factor assessment by comparing visually derived outcome scores from two-dimensional (2D) video data with dichotomized three-dimensional (3D) biomechanical variables collected using motion capture technology. A total of 41 female club volleyball athletes (14.7 ± 1.4 years) were tested and asked to perform three tasks: double-leg vertical jump (DLVJ), single-leg squat (SLS), and single-leg drop landing (SLDL). One rater was trained on the scoring criteria for the 2D data and independently scored one forward-facing and one side-facing video for each task. Risk factors identified included poor knee position, lateral trunk lean, and excessive trunk flexion/extension. In addition, 3D joint angles were calculated for the trunk and knee in the sagittal and frontal planes and converted to dichotomous variables based on biomechanical thresholds of injury risk. For comparison of 2D and 3D outcomes, percent agreement and Cohen's kappa were calculated for each risk factor individually. Overall, 2D scores were found to exhibit moderate to excellent percent agreement with 3D outcomes for trunk position (69.1–97.1%). Specifically, ipsilateral trunk lean during single-leg tasks exhibited the highest agreement (85.3–88.2%) with moderate reliability (κ = 0.452–0.465). In addition, moderate to substantial reliability was found for trunk flexion during double-leg tasks (κ = 0.521–0.653); however, an evaluation of single-leg tasks resulted in only fair reliability (κ = 0.354). Furthermore, 2D scores were not successful in identifying poor knee position as percent agreement fell below 50% for both the single-leg tasks and averaged 60% agreement across both the phases of the DLVJ. Kappa coefficients further emphasized these trends indicating no to slight concurrent validity (κ = −0.047–0.167) across tasks. Overall, these findings emphasize the potential for valid, low-cost screening tools that can identify high-risk movement patterns. Further study is needed to develop improved assessment guidelines that may be employed through visual assessment in sports environments.

## Introduction

The annual rate of anterior cruciate ligament (ACL) injuries has nearly doubled in active adolescents over the past two decades (Mall et al., 2014; Beck et al., 2017). In addition, female adolescents who participate in high-impact sports involving pivots and jumps experience ACL injuries at 4 to 6 times the rate of male athletes who participate in the same sports, with an estimated 38,000 ACL injuries annually across the United States (Toth and Cordasco, 2001; Hewett et al., 2005). As sports participation rates increase among female adolescents, many factors contribute to the increased risk of ACL injury in this population (Hewett et al., 2005; Montalvo et al., 2019). One major factor highlighted in recent literature is poor neuromuscular control at the knee during landing tasks (Hewett, 2000; Hewett et al., 2002, 2005; McLean et al., 2004; Montalvo et al., 2019). Consequently, injury prevention programs aimed at improving neuromuscular control have been studied extensively and have been shown to successfully decrease the incidence of ACL injuries, indicating elevated injury risk is modifiable (Augustsson et al., 2011; Kilic et al., 2017; Sugimoto et al., 2017). However, to the best of our knowledge, there remains limited prospective data on the validity of low-cost screening tools that can proactively identify ACL injury risk, specifically for adolescent female athletes. Furthermore, given that injury rates have been shown to vary by sport (de Loës et al., 2000; Hootman et al., 2007; Foss et al., 2018; Bram et al., 2021), screenings focused on dynamic movements specific to sports with high injury rates for female athletes may prove beneficial and more impactful for risk reduction in this population.

In volleyball, female athletes are at higher risk of ACL injury during landing tasks, such as a block jump or jump attack, potentially due to reduced knee flexion (i.e., stiff landing) and increased valgus or medial collapse of the knee (Zahradnik et al., 2020). Knee valgus during functional tasks, especially single-leg landing maneuvers, has been identified as a strong risk factor for non-contact ACL injuries (Hewett et al., 2005; Griffin et al., 2006; Munro et al., 2012; Almangoush et al., 2014; Herrington, 2014), as poor knee position during these tasks places greater demand on tissues and structures that stabilize the knee (Hewett et al., 2005). Similarly, a more erect or upright trunk position, more commonly observed in female athletes, has also been associated with increased ACL injury risk, given that it may lead to elevated landing forces (Blackburn and Padua, 2009). Conversely, a forward-flexed trunk position results in greater hip and knee flexion upon landing, reducing landing forces and mitigating ACL injury risk (Blackburn and Padua, 2009). Ipsilateral trunk lean has also been identified as a risk factor for ACL injury, as it causes the ground reaction force vector to shift laterally relative to the knee joint, increasing the load on the medial knee (Hewett et al., 2009; Sheikhi et al., 2021). Specifically, lateral trunk displacement was identified as a risk factor for ACL injury with high sensitivity and specificity in female athletes (Zazulak et al., 2007). Overall, relevant literature has shown that these neuromuscular control deficits are modifiable through neuromuscular training programs, emphasizing the importance of screenings that can proactively detect these deficits (Soligard et al., 2008; Augustsson et al., 2011; Kilic et al., 2017; Sugimoto et al., 2017). To reduce injury rates in female athletes, it is essential that athletes who demonstrate injury risk factors are identified and allowed the opportunity for retraining and exposure to risk reduction interventions.

Motion capture technology has long been regarded as the gold standard for movement assessments, but is not widely accessible because of cost and the technical training required to use the equipment (Hewett et al., 2005; McLean et al., 2005; Ekegren et al., 2009; Myer et al., 2015). Thus, the development of low-technology scoring systems to assess lower extremity alignment and trunk position has led to the popularization of visual assessments, given the ability to provide immediate feedback and quickly evaluate large groups of individuals (Stensrud et al., 2011). Although easily implemented in the community, the reliability and accuracy of visually rating movement quality remain potentially problematic, as results may vary due to inconsistent training procedures and differing levels of rating experience (Almangoush et al., 2014; Walbright et al., 2017; Lopes et al., 2018; Asgari et al., 2021). Some two-dimensional (2D) visual assessments, while found to show moderate to excellent agreement with three-dimensional (3D) measures overall, varied by individual scoring criteria specific to particular risk factors (Onate et al., 2010; Smith et al., 2012; Hanzlíková and Hébert-Losier, 2020). Furthermore, according to a systematic review performed by Lopes et al. (2018), the face validity of 2D vs. 3D measures varied by task, specifically during squatting, landing, and cutting tasks when evaluating frontal plane kinematics of the trunk and lower extremity (Lopes et al., 2018). Such discrepancies in scoring criteria and across tasks prove problematic for developing simple, reliable, and low-cost screening tools that can be employed on a large scale by coaches and clinicians. However, improving these tools is essential for making a more direct impact on the adolescent athlete population.

To the best of our knowledge, injury prevention literature has not reported the utilization of video analysis for identifying faulty movement patterns specific to adolescent female volleyball players. Previous work focused on reducing the risk of ACL injuries has highlighted the need for more proactive identification of high-risk movements present in this population (de Loës et al., 2000; Hewett, 2000; Griffin et al., 2006; Hootman et al., 2007; Beck et al., 2017; Foss et al., 2018; Bram et al., 2021). Specifically, a valid 2D video-based movement assessment that can be conducted quickly and with affordable equipment would allow for efficient, large-scale screening to more feasibly identify individuals who exhibit known musculoskeletal injury risk factors. Therefore, the purpose of this study was to assess the concurrent validity of a three-task injury risk factor assessment by comparing visually derived outcome scores with analogous biomechanical variables captured using 3D motion capture technology. Given the standardization of the current assessment that employed multiple 2D views and well-defined scoring criteria, we hypothesized that 2D measures captured across the three tasks would show substantial to almost perfect or perfect concurrent validity compared to 3D biomechanical measures.

## Methods

### Study Design and Participants

A cross-sectional study design was used and a convenience sample of 41 youth athletes was recruited from local volleyball clubs. Inclusion criteria required participants to be female volleyball athletes aged 10–18 years. One exclusion criterion was the presence of a recent musculoskeletal injury. Specifically, participants were not tested if they reported having any recent lower extremity injuries (within the past 3 months) or an orthopedic condition that would limit their ability to perform a jump or squat task. This study was approved by the local Institutional Review Board and all the participants who agreed to take part in this study provided informed assent/consent before testing. During the movement assessment, participants were asked to wear comfortable attire and personal athletic footwear.

### Data Collection

**Figure 1:**
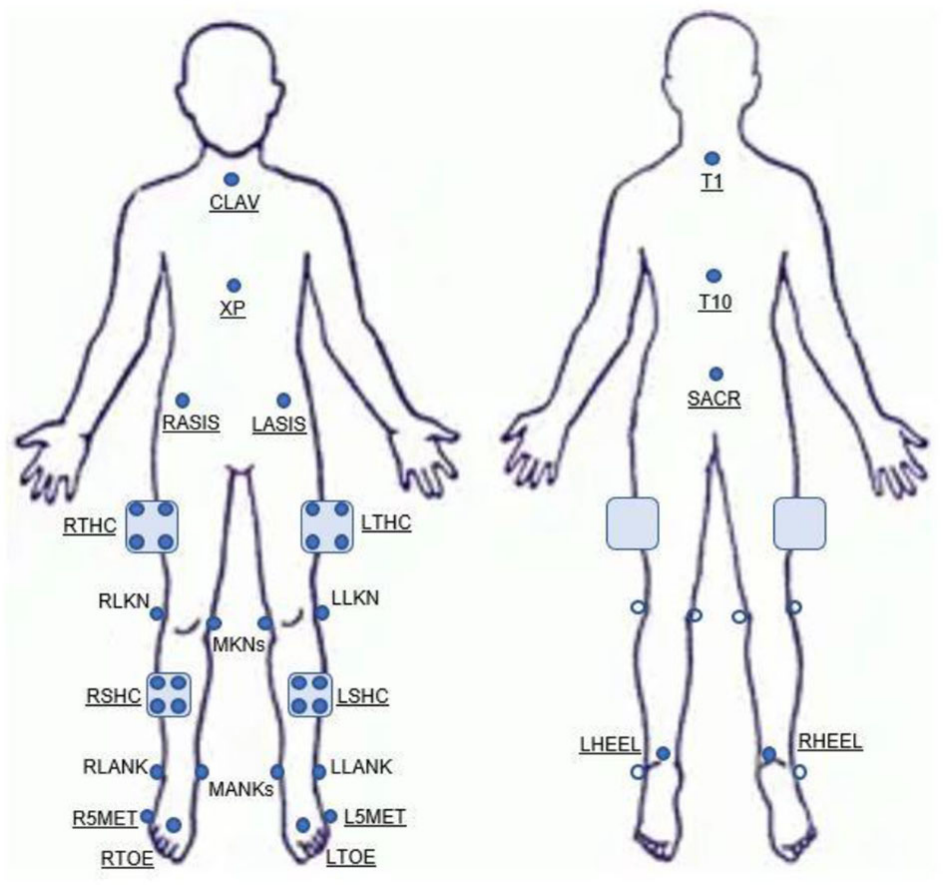
Marker set diagram.

Before the movement assessment, participants were asked to complete a brief questionnaire to capture years of experience playing volleyball and their current activity level. Activity level was determined using the Tegner Scale of 0–10, where a higher score indicates a higher activity level (Tegner and Lysholm, 1985). After recording height and weight, participants were instrumented with 21 retroreflective markers placed on specific bony landmarks and 4 clusters placed on each segment of the lower extremities (i.e., mid-thigh and mid-shank; Figure 1). 3D kinematic data were collected using a 14-camera motion capture system (Vicon Motion System Ltd., Denver, Colorado, USA), capturing at 240 Hz while participants performed a series of dynamic tasks. Simultaneously, 2D video data were collected using a single digital video camera (Sony Cyber-shot DSC-Rx10, Tokyo, Japan) positioned 36 inches high and 136 inches from the participant, capturing at 60 frames per second with 1,080-pixel quality.

Participants performed three tasks, which included a double-leg vertical jump (DLVJ), single-leg squat (SLS), and single-leg drop landing (SLDL). The DLVJ required participants to begin in a standing position and then jump as high as possible, without a preparatory step, and use their arms for momentum. To eliminate trials that were not completed correctly, errors for each task were predefined based on prior literature (Onate et al., 2010; Almangoush et al., 2014; DiCesare et al., 2014). An error was recorded if the participant failed to achieve a flight phase (i.e., leave the ground) or if they fell or stumbled upon landing. For the SLS, participants were instructed to stand on one leg with their hands on their hips and the opposite leg bent behind them, squat down as far as they could comfortably, and then return to the starting position. An error was recorded for the SLS if the participant placed the nonstance leg down to regain balance or if their hands were removed from their hips. Lastly, the SLDL required participants to stand on top of a 31-cm plyometric box with their hands on their hips, then jump down from the box landing on one leg, and hold the landing for at least 2 s. Errors for SLDL included failure to achieve the flight phase (i.e., stepping off the box rather than jumping), landing on two feet rather than one foot, removing their hands from their hips, falling or stumbling upon landing, or not sticking the landing (i.e., shuffling their landing foot). Three practice trials per task were performed to confirm the participant's understanding of the task instructions and to allow them to feel comfortable performing each task. Participants completed two attempts per task. Two attempts were collected such that video data captured on the single-camera included one trial with the participant facing forward and a second trial with the participant facing sideways. If an error occurred during the first two attempts, a third attempt was granted with the participant facing the same direction as the error trial. If the participant was unsuccessful on the third attempt, their 2D data were not scored and removed from the analysis.

### Risk Factor Assessment

**Figure 2:**
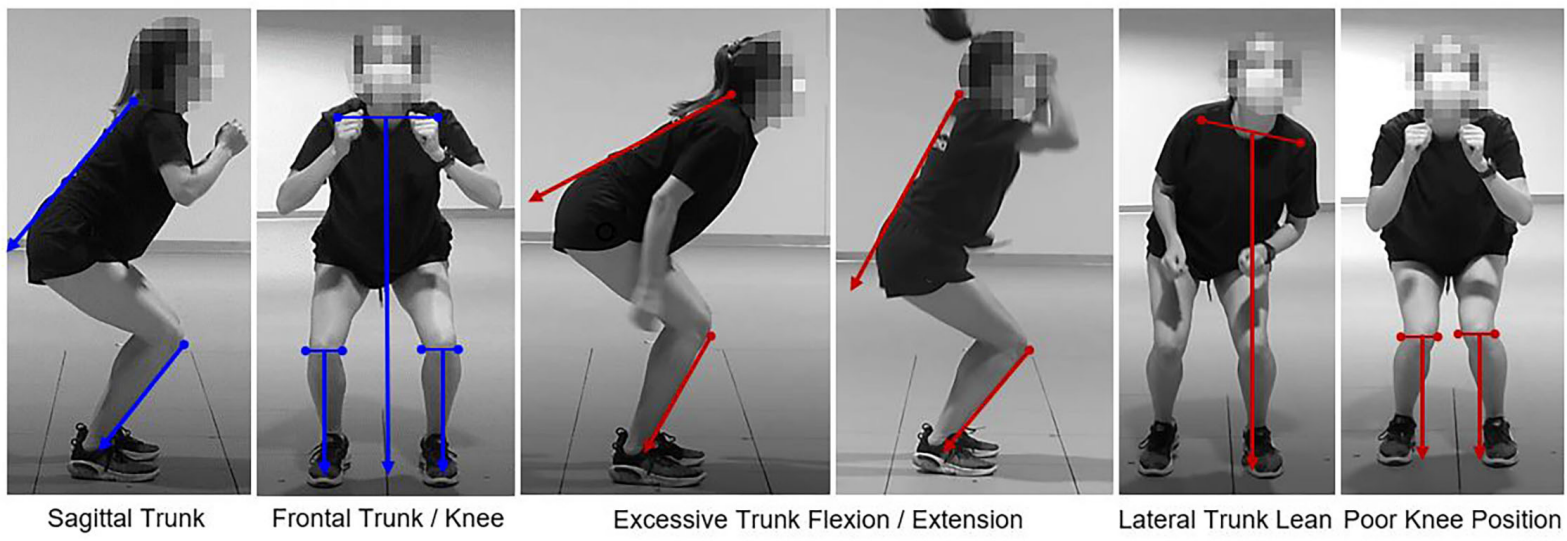
Proper alignment (left in blue) and *present* risk factors (right in red) for the double-leg vertical jump (DLVJ).

**Figure 3:**
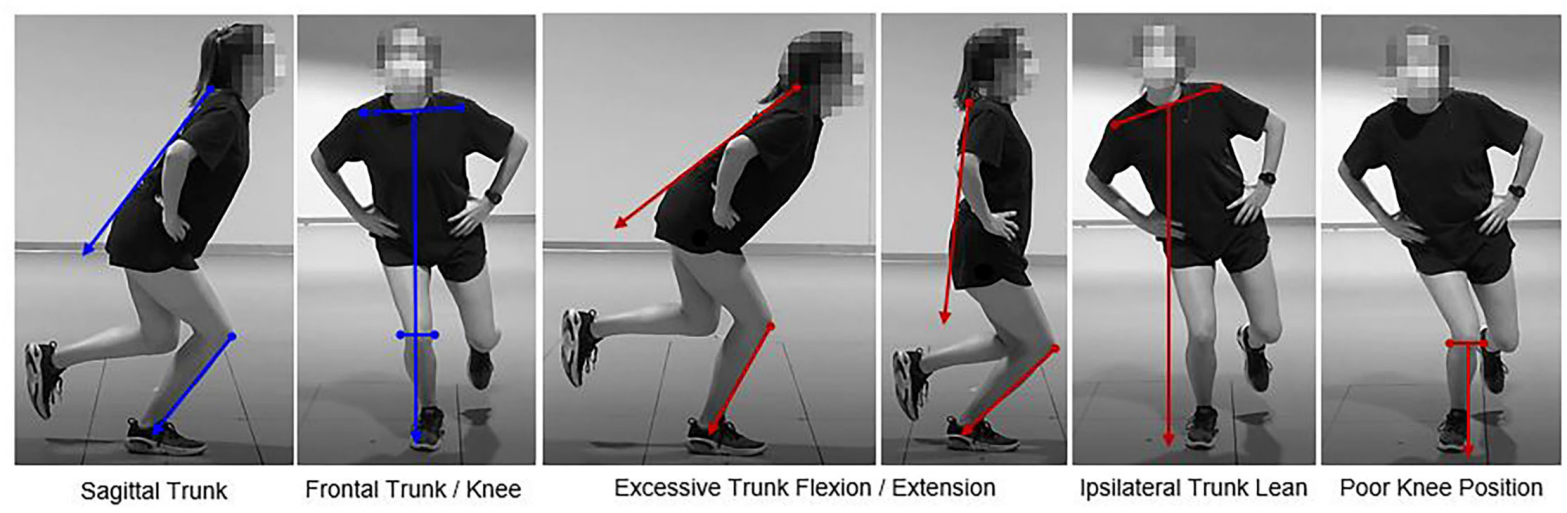
Proper alignment (left in blue) and *present* risk factors (right in red) for the single-leg tasks.

Risk factor definitions were based on relevant literature (Griffin et al., 2006; Blackburn and Padua, 2009; Ekegren et al., 2009; Wilczyński et al., 2020) and were developed by multidisciplinary clinician-scientists, namely, experienced biomechanists, physical therapists, athletic trainers, advanced practice providers, and orthopedic physicians. Risk factors were identified after data collection by reviewing the video data for each task. Risk factors included poor knee position, lateral trunk lean, and excessive trunk flexion/extension (Figures 2, 3). The 2D video was examined for each risk factor and scored as either *present* or *not present* during specific phases of each task. From the forward-facing view, poor knee position and lateral trunk lean were identified. Poor knee position was marked *present* if the center of the knee joint was inside the medial border of the shoe. Lateral trunk lean was marked *present* if the midpoint between the shoulders did not remain over the stance foot for single-leg tasks (SLS and SLDL) or if it moved away from the midpoint and closer to a knee joint during the DLVJ. Similarly, excessive trunk flexion or extension was identified from the side-facing view and marked *present* if the trunk segment did not remain parallel with the lower leg (the anterior edge of the tibia was used for reference) (Song et al., 2021). Thus, excessive trunk flexion was identified when the trunk position fell more horizontal than the lower leg and excessive trunk extension was identified as *present* when the trunk appeared more vertical than the lower leg.

Phases of interest for each task were predetermined to assess the presence of the previously defined risk factors. The loading phase for the SLS and DLVJ was defined as the period from task initiation to the cessation of knee flexion. Similarly, the landing phase for the SLDL and DLVJ was defined as the period from initial foot contact to the cessation of knee flexion. Poor knee position and lateral trunk lean were evaluated across the duration of the loading and landing phases for the DLVJ, across the loading phase of the SLS and the landing phase of the SLDL. Alternatively, excessive trunk flexion/extension was evaluated at the endpoint of the loading phase for the DLVJ and SLS and the landing phase for the DLVJ and SLDL (Table 1).

**Table 1:** Phases of interest and event definitions for each task.

**Task**	**Risk factors**	**Phases**	**Events**
DLVJ-load	Poor knee position	Loading	Initiation to cessation of knee flexion
	Lateral trunk lean	Loading	Initiation to cessation of knee flexion
	Trunk flexion/extension	End of loading	At cessation of knee flexion
DLVJ-land	Poor knee position	Landing	Initial foot contact to cessation of knee flexion
	Lateral trunk lean	Landing	Initial foot contact to cessation of knee flexion
	Trunk flexion/extension	End of landing	At cessation of knee flexion
SLS	Poor knee position	Loading	Initiation to cessation of knee flexion
	Lateral trunk lean	Loading	Initiation to cessation of knee flexion
	Trunk flexion/extension	End of loading	At cessation of knee flexion
SLDL	Poor knee position	Landing	Initial foot contact to cessation of knee flexion
	Lateral trunk lean	Landing	Initial foot contact to cessation of knee flexion
	Trunk flexion/extension	End of landing	At cessation of knee flexion

### Data Analysis

One rater with 12 years of clinical biomechanics experience was trained on the scoring criteria for the 2D data and independently scored one forward-facing and one side-facing video for each task. For all the participants, risk factors were listed as *present* or *not present* during the phases of interest and errors observed per task were documented. To analyze the 3D biomechanical data, a custom MATLAB (MATLAB 2020b, Natick, Massachusetts, USA) 6 degrees of freedom model was used to compute 3D joint angles, specifically for the trunk and knee in the sagittal and frontal planes. In addition, automated custom MATLAB codes were used to detect events during each task that correlated to the corresponding time points of interest (i.e., initiation of knee flexion, cessation of knee flexion). Given each kinematic variable calculated was a continuous output, all the variables were converted to dichotomous variables based on biomechanical thresholds of injury risk preidentified in the literature (Hunt et al., 2008; Blackburn and Padua, 2009; Fox et al., 2014; Song et al., 2021). Specifically, poor knee position was marked *present* if the knee valgus angle exceeded 4.7 and 9.1° during the single-leg task and double-leg task, respectively (Fox et al., 2014). Lateral trunk lean was marked *present* if the frontal plane trunk angle exceeded 10.0° on the ipsilateral side (Hunt et al., 2008; Song et al., 2021). Lastly, excessive trunk flexion/extension was marked *present* if the sagittal plane trunk angle fell below 15.0° (extension to a more upright trunk position) or exceeded 45.0° of forward flexion (Blackburn and Padua, 2009; Song et al., 2021).

### Statistical Analysis

Mean and SD values were calculated for each athlete's characteristic and continuous biomechanical variable and the percent of participants identified as exhibiting each risk factor for both the dichotomous 2D and 3D scores were determined. For comparison of 2D and 3D outcomes, percent agreement and Cohen's kappa (95% CI) were calculated for each risk factor individually (IBM SPSS Statistics for Windows, version 24.0, Armonk, NY, USA). Percent agreement was categorized as poor (<50%), moderate (51–79%), or excellent (≥80%) and kappa values indicated slight (0.0–0.2), fair (0.21–0.40), moderate (0.41–0.60), substantial (0.61–0.80), and almost perfect or perfect (0.81–1.0) agreement (Landis and Koch, 1977).

## Results

A total of 41 female club volleyball athletes (mean age 14.7 ± 1.4 years) were tested and average demographic, anthropometric, and sports characteristics were calculated (Table 2). Seven participants completed one or more of the tasks with an error and were removed from the analysis. 2D visual assessment of risk factors found that poor knee position was observed more frequently in the double-leg task (DLVJ-load 38.2 and DLVJ-land 54.4%; Table 3) compared to the single-leg tasks (SLS 16.2 and SLDL 10.3%). The incidence of trunk lean was relatively low across all the tasks (SLS 11.8, SLDL 5.9, DLVJ-load 5.9, and DLVJ-land 11.8%). Lastly, while excessive trunk extension was commonly seen in the single-leg tasks and DLVJ-land (SLS 39.7, SLDL 42.6, and DLVJ-land 61.8%), forward trunk flexion predominantly occurred in DLVJ-load (DLVJ-load 64.7%).

**Table 2:** Athlete characteristics.

**Variable**	**Mean (SD)**
Age (years)	14.7 (1.4)
Tegner Level (0–10)	8.6 (1.3)
Experience (years)	6.2 (2.5)
Height (cm)	169.3 (8.2)
Weight (kg)	62.0 (12.9)
BMI	21.5 (3.7)

**Table 3:** Percent risk (PR) and percent agreement (PA) for each risk factor by task.

**Risk factor by task**	**2D PR**	**3D PR**	**PA**	**kappa**	**95% CI**	
					**Lower**	**Upper**
SLS trunk flexion	17.6%	4.4%	86.8%	0.354	0.054	0.654
SLS trunk extension	39.7%	14.7%	69.1%	0.277	0.075	0.480
SLS trunk lean	11.8%	20.6%	85.3%	0.465	0.192	0.739
SLS knee valgus	16.2%	52.9%	45.6%^*^	−0.047	−0.216	0.123
SLDL trunk flexion	8.8%	0.0%^∧^	100.0%	0.000	0.000	0.000
SLDL trunk extension	42.6%	52.9%	69.1%	0.388	0.174	0.601
SLDL trunk lean	5.9%	17.6%	88.2%	0.452	0.153	0.750
SLDL knee valgus	10.3%	57.4%	44.1%^*^	−0.001	−0.129	0.127
DLVJ-load trunk flexion	64.7%	52.9%	76.5%	0.521	0.240	0.802
DLVJ-load trunk extension	0.0%	0.0%^∧^	100.0%	0.000	0.000	0.000
DLVJ-load trunk lean	5.9%	2.9%^∧^	91.2%	−0.041	−0.098	0.016
DLVJ-load knee valgus	38.2%	17.6%	64.7%^*^	0.167	−0.031	0.365
DLVJ-land trunk flexion	5.9%	2.9%	97.1%	0.653	0.025	1.281
DLVJ-land trunk extension	61.8%	38.2%	76.5%	0.554	0.312	0.796
DLVJ-land trunk lean	11.8%	2.9%^∧^	85.3%	−0.049	−0.129	0.031
DLVJ-land knee valgus	54.4%	42.6%	55.8%^*^	0.129	−0.045	0.303

Percent agreement (^*^) and percent risk values (^∧^) that were too low to obtain reliable kappa coefficients indicated with symbols.

Overall, 3D outcomes indicated a mean knee valgus angle of 4.7 (SD 3.2°), 5.7 (SD 3.2°), 6.3 (SD 3.0°), and 8.1° (SD 3.5°) during the SLS, SLDL, DLVJ-load, and DLVJ-land, respectively. The percentage of limbs that exceeded the predetermined knee valgus risk threshold was higher in the single-leg tasks compared to the double-leg task (SLS 52.9, SLDL 57.4, DLVJ-load 17.6, and DLVJ-land 42.6%; Table 3). Similar to 2D findings, sagittal trunk position evaluated at maximum knee flexion was predominantly more extended for the single-leg tasks [SLS 14.7, 26.0 (SD 10.3°); SLDL 52.9%, 14.6° (SD 9.6°)] and DLVJ-land [38.2%, 19.1° (SD 13.1°)], while forward trunk flexion was more prevalent in the DLVJ-load [52.9%, 46.3° (SD 10.8°)]. Trunk lean was not commonly exhibited during the DLVJ based on 3D outcomes [DLVJ-load 2.9, 3.6 (SD 2.1°); DLVJ-land 2.9%, 3.7° (SD 2.4°)]. However, based on predetermined thresholds, ipsilateral trunk lean was observed in 20.6 and 17.6% of participants during the SLS [6.3° (SD 5.0°)] and SLDL [5.4° (SD 5.2°)], respectively.

**Figure 4:**
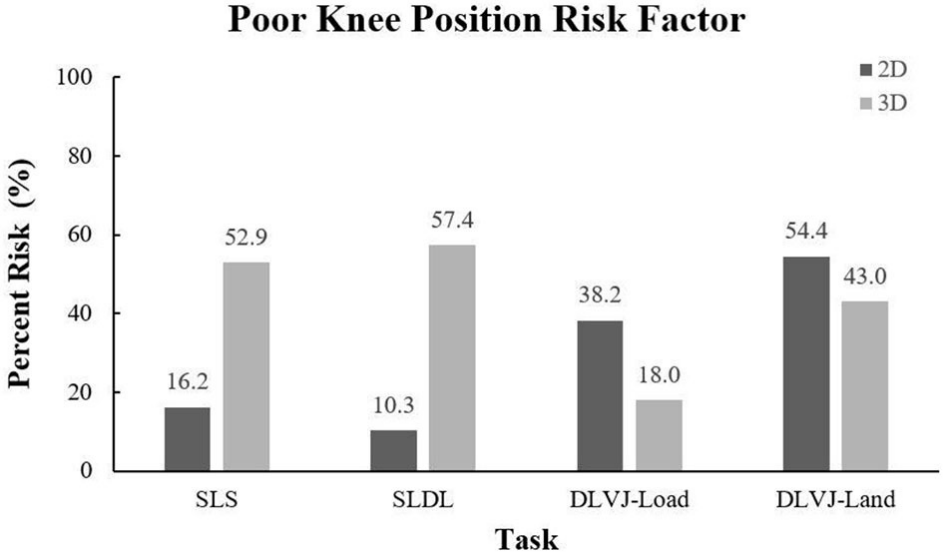
2D and 3D percent risk for the poor knee position risk factor.

The average percent agreement between the 2D scores and the 3D dichotomized variables ranged from 44.1 to 100% (Table 3). Agreement for ipsilateral trunk lean was the strongest for the single-leg tasks, with the agreement of 85.3 (SLS) and 88.2% (SLDL) with moderate reliability (κ = 0.465 and 0.452, respectively). Trunk flexion also indicated good agreement ranging between 76.5 and 100% across all the tasks, with moderate to substantial reliability during the DLVJ (load: κ = 0.521; land: κ = 0.653) and fair reliability during the SLS (κ = 0.354). Given the low incidence of trunk lean during the DLVJ (3D percent risk: 2.9%), no agreement between 2D and 3D scores was found. Similarly, excessive forward trunk flexion at the end of the SLDL landing phase and trunk extension at the end of the DLVJ loading phase were not exhibited in the 3D biomechanical assessment and, therefore, agreement with 2D scores could not be assessed. Knee valgus (or for 2D scores, knee medial to the inside of the foot) displayed the weakest agreement of the evaluated risk factors (Figure 4). There was no agreement in knee valgus for the SLS or SLDL (κ = −0.047 and −0.001, respectively). Specifically, the percent risk was determined to be higher based on 3D scores. Alternatively, during the DLVJ, 2D percent risk was higher and slight agreement was measured in loading (κ = 0.167) and landing (κ = 0.129) phases with 64.7 and 55.8% agreement, respectively.

## Discussion

The purpose of this study was to determine the concurrent validity of a three-task injury risk factor assessment by comparing 2D visual assessment scores to dichotomized 3D outcomes based on previously reported biomechanical thresholds. The assessment consisted of an SLS, SLDL, and DLVJ task and injury risk factors assessed included poor knee position, trunk lean, and excessive trunk flexion or extension during the loading and/or landing phases of each task. Overall, 2D visual assessment scores were found to exhibit moderate to excellent percent agreement with 3D outcomes for trunk position, especially ipsilateral trunk lean during single-leg tasks with an average of 87% agreement and moderate reliability. In addition, moderate to substantial reliability was found for the trunk flexion risk factor during double-leg tasks; however, an evaluation of single-leg tasks resulted in only fair reliability. Furthermore, 2D scores were not as successful in identifying knee valgus as percent agreement fell below 50% for both single-leg tasks and averaged 60% agreement across both the phases of the DLVJ. In accounting for the possibility of chance agreement, kappa coefficients emphasized these trends indicating no to slight concurrent validity.

Given the emphasis on movement screens to support injury risk reduction in adolescent athlete populations, numerous reports have investigated the agreement between 2D visual assessment and 3D biomechanical outcomes presented in this study. Specific to risk factors involving trunk position, previous 2D visual assessment studies reported a wide range of agreement compared to 3D motion analysis (Schurr et al., 2017; Straub and Powers, 2022), which have conflicting findings across assessment view and task. For example, Schurr et al. found a moderate correlation for trunk movement in the sagittal plane during a single-leg squat (Schurr et al., 2017), similar to the findings presented here. However, in contrast to the current findings, no significant correlations were found between 2D and 3D frontal plane trunk movement (Schurr et al., 2017). In addition, Kingston et al. found no agreement in trunk lean measures during a single-leg squat or double-leg vertical jump; however, they report moderate agreement between 2D and 3D measures captured during a single-leg hop task (Kingston et al., 2020). Thus, the agreement may be dependent on the task or rather the range of motion available in a single plane during a particular task. Furthermore, while DiCesare et al. reported similar trunk lean angles were exhibited across 2D and 3D measures, this agreement was dependent on the definition used to determine the 2D movement (DiCesare et al., 2014). Specifically, out of four trunk lean definitions for the 2D assessment, only one was found to agree with 3D measures. Thus, explicit risk factor definitions are essential for valid 2D assessment and are potentially a leading cause of the conflicting literature.

Unlike findings for the 2D assessment of trunk position presented here, visually identifying the presence of poor knee position, defined as the center of the knee joint collapsing medially past the inside border of the shoe, proved to be substantially more difficult across tasks, especially for the single-leg tasks. As seen in Figure 4, the risk of poor knee position was identified in considerably fewer participants compared to the 3D percent risk calculated (41.9% less on average). Alternatively, 2D percent risk was 33.1% higher in double-leg tasks compared to the single-leg scores, on average, but only 15.8% higher than the corresponding 3D percent risk. Reflecting these noted discrepancies, numerous reports evaluating the validity of 2D assessments of knee valgus during single-leg squatting and landing tasks have stated that 2D frontal plane measures alone do not reflect 3D biomechanics of the knee (Dingenen et al., 2014; Schurr et al., 2017; Lopes et al., 2018; Kingston et al., 2020). One explanation is that medial knee collapse is a combination of movements across more than one plane, namely, femoral adduction and internal rotation, anterior tibial translation, external tibial rotation, potentially ankle eversion, and knee valgus in the frontal plane (Wilczyński et al., 2020). However, Ekegren et al. claimed that specificity values were adequate for screening purposes (60–72%) and that only the sensitivity of the observational ratings was inadequate (67–87%) as raters failed to identify high-risk individuals a third of the time (Ekegren et al., 2009), similar to this study. Given the findings presented here and similar reports in the literature, 2D visual assessments of poor knee position may require a more comprehensive risk factor definition for single-leg tasks.

In contrast, Peebles et al. reported good to excellent agreement between 2D movement patterns and 3D motion capture measures (Peebles et al., 2021). Specifically, good to an excellent agreement was indicated across frontal plane knee measures for both a unilateral and bilateral landing task. Contrary to Ekegren et al. and this study, Peebles et al. utilized a more technical and complex strategy for identifying risk factors from 2D video data, commonly reported in the literature (DiCesare et al., 2014; Dingenen et al., 2014; Peebles et al., 2021). This technique requires raters to extract a still image from the video data at the time point of interest and manually select landmarks on the individual's body (e.g., joint center) used to calculate joint angles. While this technique has indicated high agreement with 3D motion analysis and is overall less expensive and more feasible compared to 3D motion analysis, the training, time, and equipment necessary to process movement screens would not prove ideal for coaches or clinicians in a sports environment (e.g., practice, development camp). Procedures for the 2D visual assessment described in this study only require a single video camera, a sturdy platform for the SLDL, and a rater trained on the outlined risk factor definitions. In addition, the definitions for scoring the movement assessment were developed such that a clinical or biomechanical background would not be necessary. Thus, these findings highlight the potential for coaches and athletic trainers to be trained to conduct the 2D assessment presented here to identify high-risk athletes under their supervision. Further study is needed to improve the identification of poor knee position.

### Limitations

It is also important to note that conflicting results in the literature may be due to the populations tested. Specifically, the previously referenced articles conducted movement assessments on groups of single-sport, adolescent female athletes (Ekegren et al., 2009; DiCesare et al., 2014), recreationally active young adults (Schurr et al., 2017; Peebles et al., 2021), and elite college-aged female athletes who played soccer, handball, or volleyball (Dingenen et al., 2014). Validity reports have also evaluated 2D visual assessments in specific patient populations (Kingston et al., 2020). Similar to the previous study, one limitation of this study is that validity of the 2D visual assessment was evaluated in a homogeneous group of female volleyball athletes that all participated at a competitive club level. Given that injury rates and risk factors have been shown to differ by sport, age group, and prior injury (de Loës et al., 2000; Hootman et al., 2007; Foss et al., 2018; Bram et al., 2021), it is important to understand the constraints of the results presented here and in the literature. Future studies will focus on extending validity testing to male athletes, alternative sports, and various competition levels. Another limitation of the current movement assessment is that it lacks an evaluation of hip movement during the three tasks. Increased hip adduction is a strong contributor to dynamic knee valgus and, therefore, ACL injury risk (Krosshaug et al., 2007; Powers, 2010; Scholtes and Salsich, 2017). Thus, the poor knee position risk factor included here may need redefining to include hip landmarks or an additional risk factor for the hip that may need to be evaluated in the future studies. The latter is supported by multiple reports in which 2D hip joint angles exhibited moderate to strong correlations with 3D measures (Scholtes and Salsich, 2017; Kingston et al., 2020).

In addition, an inherent limitation to comparing 2D and 3D data is that the two motion analysis systems did not capture data at the same rate. Thus, it is possible that risk factors identified at a specific frame in the 2D video were not identified at the same time point in the 3D trial. As the primary goal of this study was to evaluate the concurrent validity of the visual assessment, which requires the rater to manually identify specific time points for evaluation, this was considered an acceptable and minor limitation to this study. Similarly, the sagittal and frontal views were not captured in the same trial, which potentially increased data variability. While a single camera view was chosen specifically such that implementation in the field would require less equipment and technical setup to ensure video data is synced, this is recognized as a minor limitation of this study. Last, given that 2D video and 3D motion capture data were captured simultaneously, the retroreflective markers needed for motion capture were visible in all the 2D video trials. Specifically, markers were positioned on the medial and lateral femoral epicondyle, lateral malleolus, and the jugular notch, all of which may have potentially helped the rater visually inspect the knee joint center, lower leg orientation, or midpoint between the shoulders. While the rater was instructed to disregard the retroreflective markers, the inclusion of the marked landmarks in the 2D video data is a minor limitation of this study as it would not be available for future utilization of the movement screen.

## Conclusion

Overall, these findings emphasize the potential for less expensive, more feasible screening methods that are valid compared to more advanced methods of identifying high-risk movement patterns. However, additional study is needed to improve upon the scoring criteria. Given poor agreement was found for the knee position risk factor presented here, future studies will focus on adjusting the risk factor definition and, thus, scoring criterion, by developing a more explicit definition using easily discernible landmarks to better align with the biomechanical movement pattern. The goal of future studies should not be to increase the complexity of assessing injury risk, but rather to develop clear assessment guidelines that may be employed through visual assessment in sports environments.

## Data Availability Statement

The raw data supporting the conclusions of this article will be made available by the authors, without undue reservation.

## Ethics Statement

The studies involving human participants were reviewed and approved by University of Texas Southwestern Institutional Review Board. Written informed consent to participate in this study was provided by the participants' legal guardian/next of kin.

## Author Contributions

SU, AE, and AL led participant recruitment efforts and conducted data collection. SU and AE organized the database and wrote the first draft of the manuscript. SU performed the statistical analysis. AL wrote a section of the manuscript. All authors contributed to conception and design of the study and contributed to manuscript revision, read, and approved the submitted version.

## Conflict of Interest

The authors declare that the research was conducted in the absence of any commercial or financial relationships that could be construed as a potential conflict of interest.

## Publisher's Note

All claims expressed in this article are solely those of the authors and do not necessarily represent those of their affiliated organizations, or those of the publisher, the editors and the reviewers. Any product that may be evaluated in this article, or claim that may be made by its manufacturer, is not guaranteed or endorsed by the publisher.
